# Diagnostic accuracy of fluorescence flow-cytometry technology using Sysmex XN-31 for imported malaria in a non-endemic setting

**DOI:** 10.1051/parasite/2022031

**Published:** 2022-05-31

**Authors:** Stéphane Picot, Thomas Perpoint, Christian Chidiac, Alain Sigal, Etienne Javouhey, Yves Gillet, Laurent Jacquin, Marion Douplat, Karim Tazarourte, Laurent Argaud, Martine Wallon, Charline Miossec, Guillaume Bonnot, Anne-Lise Bienvenu

**Affiliations:** 1 Service de Parasitologie et Mycologie Médicale, Groupement Hospitalier Nord, Hospices Civils de Lyon69004 LyonFrance; 2 Université de Lyon, Université Lyon 1, CNRS, INSA, CPE-Lyon, ICBMS, UMR 524669100 VilleurbanneFrance; 3 Service des Maladies Infectieuses et Tropicales, Hôpital de la Croix-Rousse, Hospices Civils de Lyon69004 LyonFrance; 4 CIRI Équipe PH3ID – INSERM – U1111– UCBL Lyon 1 – CNRS – UMR5308 – ENS de Lyon69007 LyonFrance; 5 Service d’accueil des urgences, Hôpital de la Croix-Rousse, Hospices Civils de Lyon69004 LyonFrance; 6 Service de Réanimation et Urgences Pédiatriques, Hôpital Femme-Mere-Enfant, Hospices Civils de Lyon69500 LyonFrance; 7 Service d’accueil des urgences, Hôpital Edouard Herriot, Hospices Civils de Lyon69008 LyonFrance; 8 Service d’accueil des urgences, Hôpital Lyon Sud, Hospices Civils de Lyon69310 LyonFrance; 9 Université de Lyon, Université Claude Bernard Lyon 1, HESPER EA 742569008 LyonFrance; 10 Hospices Civils de Lyon, Hôpital Edouard Herriot, Service de Médecine Intensive-Réanimation69008 LyonFrance; 11 Service Pharmacie, Groupement Hospitalier Nord, Hospices Civils de Lyon69004 LyonFrance

**Keywords:** Malaria, Plasmodium, Diagnostic accuracy, Sysmex XN-31, RDT, LAMP, Microscopy, PCR

## Abstract

Malaria diagnosis based on microscopy is impaired by the gradual disappearance of experienced microscopists in non-endemic areas. Aside from the conventional diagnostic methods, fluorescence flow cytometry technology using Sysmex XN-31, an automated haematology analyser, has been registered to support malaria diagnosis. The aim of this prospective, monocentric, non-interventional study was to evaluate the diagnostic accuracy of the XN-31 for the initial diagnosis or follow-up of imported malaria cases compared to the reference malaria tests including microscopy, loop mediated isothermal amplification, and rapid diagnostic tests. Over a one-year period, 357 blood samples were analysed, including 248 negative and 109 positive malaria samples. Compared to microscopy, XN-31 showed sensitivity of 100% (95% CI: 97.13–100) and specificity of 98.39% (95% CI: 95.56–100) for the initial diagnosis of imported malaria cases. Moreover, it provided accurate species identification as*falciparum*or non-*falciparum*and parasitaemia determination in a very short time compared to other methods. We also demonstrated that XN-31 was a reliable method for patient follow-up on days 3, 7, and 28. Malaria diagnosis can be improved in non-endemic areas by the use of dedicated haematology analysers coupled with standard microscopy or other methods in development, such as artificial intelligence for blood slide reading. Given that XN-31 provided an accurate diagnosis in 1 min, it may reduce the time interval before treatment and thus improve the outcome of patient who have malaria.

## Abstract (fr)

Le diagnostic du paludisme basé sur la microscopie est rendu difficile par la disparition progressive des microscopistes expérimentés en zone non-endémique. À côté des méthodes conventionnelles, la technique de cytométrie de flux en fluorescence utilisant le Sysmex XN-31, un automate d’analyse hématologique, a été enregistrée pour participer au diagnostic du paludisme. L’objectif de cette étude prospective, monocentrique et non-interventionelle était d’évaluer la précision diagnostique du XN-31 pour le diagnostic initial et le suivi des cas de paludisme d’importation en comparaison des tests de référence dont la microscopie, l’amplification isothermale en boucle, et des tests de diagnostic rapide. Durant une période d’un an, 357 échantillons de sang ont été analysés, dont 248 négatifs et 109 positifs pour le paludisme. En comparaison de la microscopie, le XN-31 a montré une sensibilité de 100 % (95 % CI : 97.13-100) et une spécificité de 98.39 % (95 % CI : 95.56-100) pour le diagnostic initial des cas de paludisme d’importation. De plus, l’identification des espèces*falciparum*et non-*falciparum*ainsi que la parasitémie ont été précises dans un temps très court en comparaison des autres méthodes. Nous avons aussi démontré que le XN-31 était une méthode fiable pour le suivi des patients à J3, J7 et J28. Le diagnostic du paludisme peut être amélioré en zone non-endémique par l’utilisation d’automates d’hématologie spécialisés, associés à la microscopie standard ou d’autres méthodes en développement telle que l’intelligence artificielle appliquée à la lecture des lames de sang. Dans la mesure où le XN-31 produit un diagnostic précis en une minute, cela peut réduire le délai avant le traitement et donc améliorer l’issue pour les patients souffrant de paludisme.

## Introduction

Malaria is a life-threatening disease caused by the protozoan parasite*Plasmodium*transmitted through the bite of an infected*Anopheles*mosquito. In 2020 there were more than 240 million infections with an estimated 627,000 deaths reported globally [38]. The countries with the highest burden are located in Africa (95%), followed by South-East Asia (2%) and the Eastern Mediterranean region (2%). Five*Plasmodium*species are regularly identified in infected humans:*Plasmodium falciparum*(*P. falciparum*),*Plasmodium vivax*,*Plasmodium ovale curtisi*,*Plasmodium ovale wallikeri*(while the distinction between those two species is uncommon), and*Plasmodium malariae*[8,21]. Among the zoonotic species,*Plasmodium knowlesi*is the most frequently observed in humans [10].

Currently, France ranks first for the number of imported malaria cases per country followed by Great Britain, the United States, and Italy [14]. Patients who are diagnosed with malaria are mainly new immigrants or residents contracting malaria during family visits in their home countries. In most imported cases, patients come from Sub-Saharan Africa and the predominantly identified parasite species are*P. falciparum*(86.5%), followed by*P. ovale*(6.8%),*P. vivax*(2.5%),*P. malariae*(2.2%), and multiple*Plasmodium species*(1.3%) [14]. Early and accurate diagnosis of imported malaria cases is fundamental for successful treatment of the infection, the stewardship of drug resistance, and malaria surveillance [3]. One pillar in the diagnostic workflow is microscopy of a stained blood smear, which is time-consuming and requires an experienced microscopist [1,5,39]. Rapid diagnostic antigen tests (RDTs), loop-mediated isothermal amplification (LAMP) and polymerase chain reaction (PCR) can support, but not replace, microscopy [28,39]. Considering the small number of imported malaria cases diagnosed each year in each diagnostic facility, it is a challenge for non-endemic countries to maintain a good level of diagnostic capacity [15,16]. Thus, alternative diagnostic methods are needed in our healthcare facilities to ensure the most reliable diagnosis of imported malaria cases [4].

Haematology analysers have been used for at least a decade for malaria diagnosis [7,11,23,26,29,33]. The XN-31 is an automated haematology analyser that obtained CE marking in April 2019 to support malaria diagnosis in whole blood samples [27,35,36,40]. Using fluorescence flow cytometry (FFC) technology, it quantifies malaria-infected red blood cells (MI-RBC) in human blood, i.e., RBCs containing nucleic acids [12,13]. Together with a complete blood count (CBC), it gives a qualitative result as well as quantification of MI-RBC per microliter (μL), and as a percentage of infected red blood cells (MI-RBC%). This analyser thereby allows for accurate and standardised detection of malaria parasites in human blood as it provides reliable results 24/7, regardless of the availability of a malaria-experienced professional [17,22,31].

The first objective of this study was to evaluate the diagnostic accuracy of the XN-31 automated haematology analyser (sensitivity, specificity, negative predictive value, and positive predictive value) for the initial diagnosis and/or follow-up of imported malaria cases compared to the reference malaria tests including RDT, LAMP, and microscopy [32]. The secondary objectives were to compare the*Plasmodium*species identification and parasitemia assessed by XN-31 to those performed by microscopic examination of the thin smear, and to study the repeatability over time of the XN-31 method after blood storage under different conditions.

## Materials and methods

### Ethics

This was a non-interventional study, without any additional procedures. Data were collected during routine patient care. Provided data were centrally checked for completeness, plausibility, and integrity before analysis. To ensure reproducibility and completeness of data extraction, an excel spreadsheet (Microsoft Corp., Redmond, WA, USA) compiling all variables to be extracted was used.

This research involved anonymised data sets in which personal identifiers were permanently and completely removed from data, meaning that the data can no longer be associated with an individual in any manner. Anonymisation and removing of protected health information from clinical narratives were performed according to the European Textbook on Ethics in research (http://ec.europa.eu/research/swafs/pdf/pub_archive/textbook-on-ethics-report_en.pdf). Electronic records were stored and processed in compliance with the conventions of the French National Committee for Data Protection and Freedom of Information. Ethical clearance was obtained from Lyon University hospital. The following data were collected using hospital databases: malaria final diagnosis based on RDT, LAMP and microscopic test, date of sampling at admission and follow-up if any, and drug used for malaria treatment.

### Study design

This prospective, cross-sectional, monocentric, non-interventional study, was conducted in Lyon University Hospital over a period of 12 months. The Standards for Reporting Diagnostic accuracy studies (STARD 2015) hosted by the Enhancing the Quality and Transparency of Health Research (EQUATOR) network were used as methodological support [16,17].

### Participants

Eligibility criteria were either suspicion of non-severe imported malaria based on fever and history of travel to a malaria endemic area or follow-up of a malaria-positive case. A venous blood sample was collected for malaria diagnosis on two EDTA tubes and rapidly transferred to the diagnostic facility at room temperature. All patients who were suspected of having malaria or followed up after a malaria-positive diagnosis were assessed for eligibility. According to the requirements for confirmatory diagnostic accuracy studies, sensitivity and specificity were considered co-primary endpoints, and the prevalence of the target population was taken into account [34]. The sample size calculation [6] was based on an expected sensitivity and specificity of XN-31 of 95% and 95%, respectively compared to microscopy, a prevalence of the disease of 20% (based on the local epidemiology in 2020 and previous years [32]), with a 95% confidence interval and 5% error. The recommended sample size for sensitivity was 365 and for specificity 92. The final sample size was the largest number (*n* = 365 participants).

### Routine diagnostic methods

LAMP (Alethia Malaria DNA amplification assays, Meridian Bioscience Inc., Cincinnati, OH, USA) and RDT (CareStart™ Malaria Pf/PAN (HRP2/pLDH) Ag Combo RDT, Access Bio Inc., Somerset, NJ, USA) were performed as screening methods according to the manufacturer’s specifications on fresh blood samples [32]. If one of these screening tests was positive, microscopic examinations of thin or thick smears were then carried out for identification and numeration of*Plasmodium*species. Thin and thick blood stained smears (Diff-Quick and Giemsa stains, respectively) were performed in compliance with the national guidelines for malaria diagnosis and certification requirements of the National Certification Program. Proficiency testing and external quality assessment for microscopy were used once a week and bi-monthly, respectively. They were read by two independent trained and certified biologists and checked by a senior microscopist. Microscopic examination allowed for the identification of*Plasmodium*species and the determination of parasitaemia, recorded as the number of infected red blood cells (RBCs) with asexual parasites compared to non-infected RBCs. Malaria microscopic diagnosis was considered to be negative if no parasites were found in 100 microscopic fields of 200 red blood cells for thin smears and 25 microscopic high power fields for thick smears. Cases of discrepancy between XN-31 and microscopic or molecular testing were resolved by a real-time PCR test as described [25].

### Sysmex XN-31 diagnosis method

The same fresh blood samples were tested with the Sysmex XN-31 analyser, according to the manufacturer’s recommendations [35,37]. Two study sub-groups were defined: samples collected at admission of the patients were included in the “inclusion-group” and samples collected during the follow-up at days 3, day 7, day 28 or any other day after positive malaria diagnosis were included in the “follow-up” group. A repeatability study over time based on the MI-RBC count was conducted on two blood samples from eight different patients randomly selected, kept either at room temperature (20–25 °C) or at 4 °C, and tested in triplicate after 24, 48 and 72 h.

## Results

**Figure 1:**
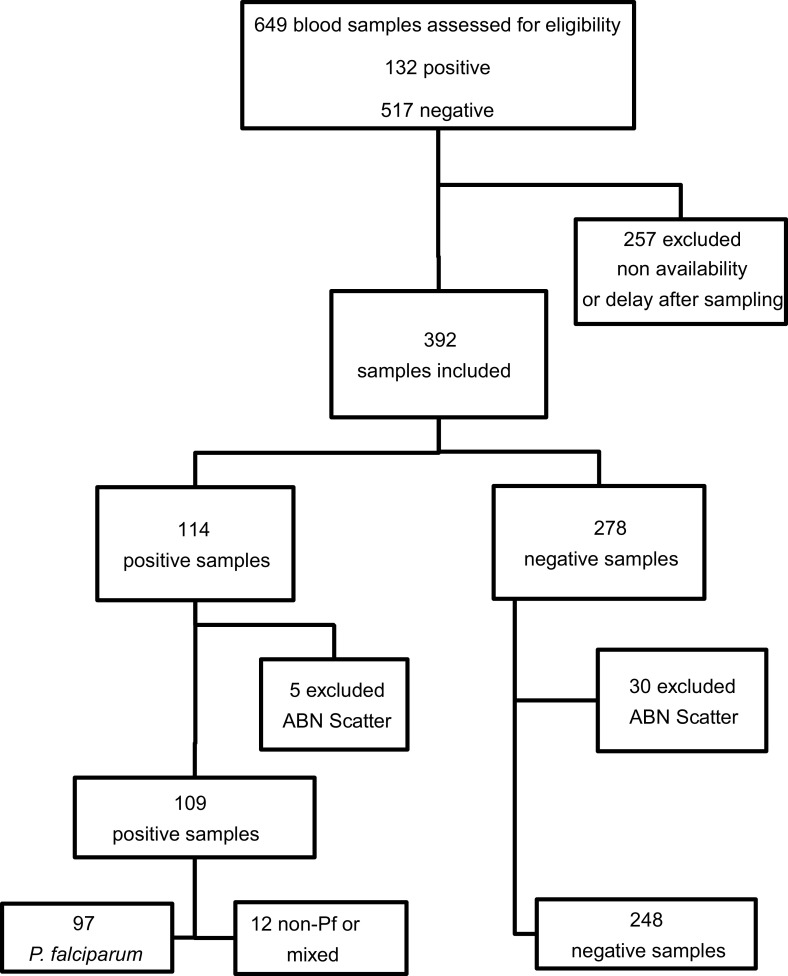
Study flow chart.

During the 12-month study period, 296 patients (age median = 36 [range 1–89]; sex ratio M/F: 1.70) were suspected of having non-severe malaria or followed up after a positive malaria diagnosis, leading to 392 blood samples eligible for inclusion. Due to MI-RBC Abnormal Scattergrams according to the XN-31 analyser, 5 positive and 30 negative samples were excluded, according to the manufacturer’s recommendations. The final study sample size was 357, including 109 positive and 248 negative samples. Among them, 288 blood samples were collected on admission and 69 during patient follow-up. Among the 109 malaria positive samples, most of them were mono-infections caused by*P. falciparum*(*n* = 97), followed by*P. malariae*(*n* = 4),*P. ovale*(*n* = 2), and*P. vivax*(*n* = 1). Five of them were mixed infections of*P. falciparum*and*P. malariae*(Fig. 1).

The first objective was to evaluate the diagnostic accuracy of the XN-31 automated haematology analyser compared to microscopy, LAMP, and RDT. According to the analysis of all included samples (*n* = 357), XN-31 showed very high sensitivity of 100% (95% CI: 97.13–100) and specificity of 98.39% (95% CI: 95.56–100) compared to microscopy (Table 1). The negative and positive predictive values of XN-31 compared to microscopy were 100% and 96.46%, respectively. These performances were slightly lower when compared to LAMP and RDT, probably because of the better specificity of XN-31 for samples included during the follow-up.

**Table 1:** (A) Diagnostic performances of Sysmex XN-31 compared to microscopy (thin or thick slides). All the blood samples (357 samples) included were used for this analysis (samples at admission and samples during the follow-up). (B) Diagnostic performances of Sysmex XN-31 compared to microscopy (thin or thick slides), Real-time PCR, LAMP (Meridian) and Rapid Diagnostic Test (CareStart) limited to the samples collected at admission (288 samples).

		Microscopy		LAMP		RDT	
		Positive	Negative	Positive	Negative	Positive	Negative
A							
XN 31	Positive	109	4	110	3	111	2
	Negative	0	244	12	232	10	234

Sensitivity	% (95% CI)	100 (97.13–100)		96.16 (93.40–98.92)		91.74 (88.87–94.37)	
Specificity	% (95% CI)	98.39 (95.56–100)		98.72 (95.88–100)		99.15 (96.30–100)	
PPV	% (95% CI)	96.46 (93.69–99.22)		97.35 (94.55–100)		98.23 (95.41–100)	
NPV	% (95% CI)	100 (97.13–100)		95.08 (92.35–97.80)		95.9 (93.03–98.65)	
*X*^2^		*p* < 0.001		*p* < 0.001		*p* < 0.001	

B							
XN 31	Positive	97	0	96	1	95	2
	Negative	1	190	5	186	4	187

Sensitivity	% (95% CI)	98.98 (96.14–100)		95.05 (92.32–97.78)		95.96 (93.21–98.71)	
Specificity	% (95% CI)	100 (97.13–100)		99.47 (96.62–100)		98.94 (96.10–100)	
PPV	% (95% CI)	100 (97.13–100)		98.97 (96.13–100)		97.94 (95.13–100)	
NPV	% (95% CI)	99.48 (96.62–100)		97.38 (94.59–100)		97.91 (95.10–100)	
*X*^2^		*p* < 0.001		*p* < 0.001		*p* < 0.001	

PPV: positive predictive value; NPV: negative predictive value.

Using only the samples collected at admission (*n* = 288), XN-31 showed sensitivity and specificity of 98.98% and 100%, respectively. In this sub-group, the VPN and VPP were 99.48% and 100%, respectively.

All the*P. falciparum*samples were correctly identified as*P. falciparum*by XN-31. All but one of the non-falciparum samples (5/6) were detected as “malaria: others” by XN31, leading to an excellent distinction between*P. falciparum*and other species. This identification was confirmed by real-time PCR as described (data not shown). One sample with*P. malariae*(RT-PCR confirmed) with low parasitaemia of 0.01% (0.0294 MI-RBC %) did not lead to species identification (“UNC?”). This sample showed 85% gametocytes and the patient had already received antimalarial treatment for two days. Considering the low parasitaemia, the high ratio of gametocytes/trophozoites, and the impact of treatment on parasite morphology, the “UNC?” comment was considered acceptable.

Interestingly, 5/5 mixed infections (*P. falciparum + P. malariae*) were also detected by XN-31, with a double indication of “*P. falciparum”*and “others” species. The mixed infections were confirmed by RT-PCR (data not shown). Additionally, four samples collected during the recrudescence episode from D28 to D34 of a patient presenting*falciparum*malaria and treated with artemether-lumefantrine on admission, and then with quinine IV during the recrudescence episode, were qualified as “*P. falciparum* + others” and “others”, while confirmed as*P. falciparum*using real-time PCR. This discrepancy may be attributed to the abnormal morphology of parasites after treatment as confirmed by microscopy. Gametocytes appeared during the last three days of the follow-up after quinine and were correctly detected by XN-31.

**Figure 2:**
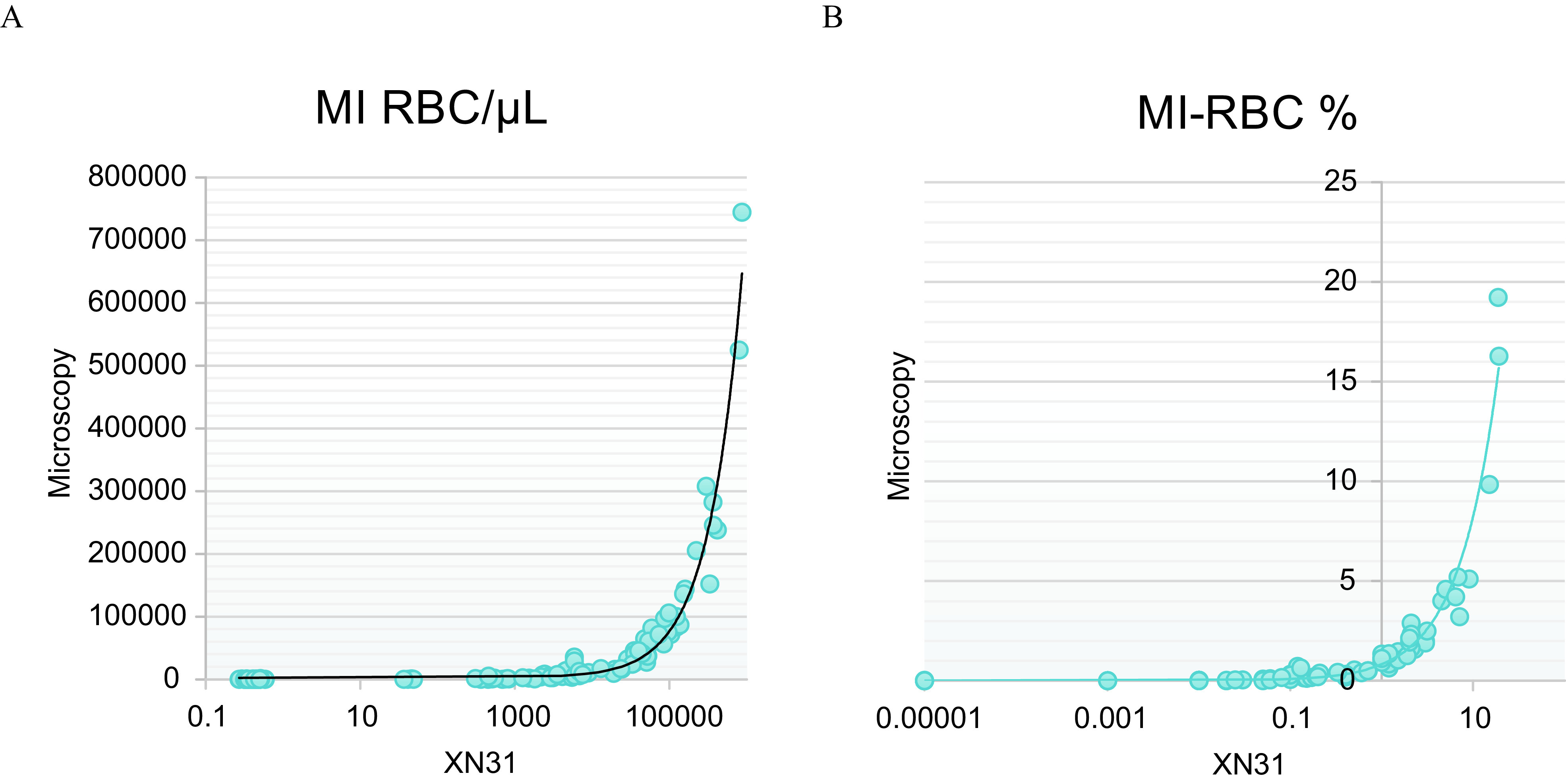
(A) Comparison of blood parasitaemia obtained with XN-31 (MI-RBC/μL) and by microscopic examination of stained thin blood smears (parasites/μL). (B) Comparison of blood parasitemia obtained with XN-31 (MI-RBC %) and by microscopic examination of stained thin blood smears as a percentage of infected red-blood-cells.

One of the secondary objectives was to compare the parasitaemia assessed by XN-31 and the parasitaemia calculated after microscopic examination of a stained thin blood smear. The correlation between XN-31 and microscopy was*R* ^2^ = 0.966, which can be considered excellent, based on the inherent uncertainty of a microscopic examination (Fig. 2).

Another secondary objective was to study the repeatability over time of the XN-31 method after blood storage under two different conditions. The repeatability study was based on the mean of MI-RBC counts on admission (H0), H24, H48, and H72. All tests were repeated three times. There was no significant difference in MI-RBC count between results of samples kept at room temperature and at 4 °C from H0 to H72 for all the samples, except one (sample 5) presenting unexplained increased values at H48 at 4 °C that disappeared at H72 (Table 2).

**Table 2:** Repeatability study of XN-31. Repeated measurements of malaria-infected red blood cells (MI-RBC) on inclusion and after storage of the blood sample (vacutainer) for 24 (H24), 48 (H48), and 72 (H72) hours. For each blood sample, one tube was stored at room temperature (20–25 °C,) and one was kept at 4 °C. The two tubes with different storage conditions were tested at 24, 48, and 72 h and the mean of triplicate measurements of the number of parasites/μL (MI-RBC) was recorded (grey line: room temperature; white line: 4 °C). Eight patients were included in this study, with initial parasitaemia ranging from 2.4 to 288.3 parasites/μL.

	Inclusion	H24	H48	H72
	Mean MI-RBC (parasites/μL): Storage at room temperature			
Samples	Mean MI-RBC (parasites/μL): Storage at 4 °C			
1	2444	2482	2343	2441
	2445	2368	2454	2467
2	2438	2399	2377	2527
	2429	2462	2525	2577
3	2947	1884	1719	1693
	3836	1912	2083	2186
4	5496	6908	5313	8341
	5712	5481	5561	5466
5	5524	4348	4029	3939
	5591	5028	14,655	3417
6	10,767	10,660	10,702	10,840
	10,726	10,663	10,692	10,898
7	205,239	206,403	205,568	202,832
	204,969	204,864	202,806	204,232
8	282,352	281,880	280,875	278,894
	282,352	278,069	279,877	280,325

## Discussion

During the 12-month study period, 357 blood samples were studied, including 109 positive and 248 negative samples. This prospective study was conducted in Lyon University hospital during the COVID-19 pandemic that led to a dramatic reduction in travel to and from malaria endemic areas, and consequently to a reduced number of suspected and confirmed malaria cases. The number of malaria cases decreased by more than 80% during 2020 and the first 3 months of 2021. Later in 2021, the number of travelers increased again significantly, allowing us to include more patients, although the number of included travelers from Asia and South America remained low.

The objective of this study was to test the XN-31 hematology analyser method for malaria diagnosis in routine conditions. To this end, data were compared to the standard diagnostic procedure which includes a molecular test (LAMP) and an antigenic test (RDT) for screening, and microscopic examination of thin and thick blood smears for parasite identification and parasitaemia determination.

The results for specificity and sensitivity (100% (95% CI: 97.13–100) and 98.39% (95% CI: 95.56–100), respectively) allowed us to conclude that the XN-31 haematology analyser was as effective as conventional methods for the screening of patients who were suspected of having malaria. While almost 10% of the tested samples were excluded due to an abnormal scattergram, as required by the study protocol, all these 35 samples (30 negative and 5 positive) showed correct malaria results. For patients who were followed up after a positive malaria diagnosis, few false-positive and false-negative results were found, compared to LAMP or RDTs. This was attributed to the persistence of circulating*Plasmodium*DNA or antigens during the patient’s follow-up, which are in fact not relevant for active malaria infection. In fact, the standard screening methods (molecular tests such as LAMP and antigenic tests such as RDT) frequently remained positive for 2–4 weeks post-infection, while parasite clearance was obtained [9]. Interestingly, when compared to microscopy, XN-31 did not yield false-positive results during patient follow-up at days 3, 7, or 28. Taken together, these data demonstrate that XN-31 could be more efficient for these days of follow-up in patients who received antimalarial treatment, compared to screening methods. This provides a major advantage for XN-31 in clinical practice, as well as during clinical trials of drug efficacy.

Similarly, XN-31 was found to be highly correlated to microscopy for species identification and parasitaemia assessment. Moreover, based on the repeatability study, there was no impact on XN-31 results of storage temperature (room temperature or 4 °C), nor of the time interval of 72 h for blood sample analysis.

Apart from its excellent technical characteristics for initial malaria diagnosis or follow-up, XN-31 has excellent practicability, and while it requires specific training, its accessibility is straightforward for non-expert users of haematology devices. When the machine is ready for the working day, the time to a result including parasitaemia is very short (within a minute), whereas RDT and LAMP as screening methods require 25 and 45 min, respectively to obtain a result without a parasitaemia count. The short timeframe to obtain a malaria result with XN-31 is a major advantage compared to other screening methods, considering that the clinical prognosis is improved with early diagnosis and treatment.

In view of the excellent performance and practicability of XN-31, we need to consider the place of XN-31 in our malaria diagnostic strategy. Some guidelines [20] from non-endemic countries recommend the use of RDTs and molecular tests for initial screening, followed by light microscopy for diagnostic confirmation, species identification, and parasitaemia count. The success of XN-31 in both screening steps and parasitaemia assessment make it a game changer in the process of imported malaria diagnosis. The majority of laboratories in non-endemic areas receive a low number of suspected malaria cases, and among those, the positivity rate is low. This epidemiological context underpins the need for a screening test that presents an excellent negative predictive value. The malaria LAMP test is known to reach this goal and its deployment among diagnosis sites, including expert sites, has increased in recent years. However, alongside the high negative predictive value, the LAMP test has some major drawbacks, including the need for sample preparation, a 40 min reaction time, the occurrence of “non-valid” results, the lack of species identification, and the lack of parasitaemia counts [30]. XN-31 is a major competitor for these essential requirements allowing for a rapid and effective medical decision and treatment linked to favourable outcomes of malaria cases. Furthermore, XN-31 also provides a detailed blood cell count at the same time, completing the biological profile of malaria infection [31]. Clearly, XN-31 could replace LAMP and RDTs as a screening test for imported malaria cases. Mid 2020, XN-31 was approved by the Japan Pharmaceuticals and Medical Devices Agency (PMDA) and included in the diagnostic flowchart by the Japanese Societies of Tropical Medicine, Parasitology and Clinical Parasitology [19]. It can be supposed that scientific societies of other non-endemic countries will rapidly approve this tool.

Compared to microscopy, XN-31 also has value regarding its capability to efficiently assess parasitaemia. Importantly, expert microscopists are not always available at the diagnostic facilities and expertise in malaria diagnosis is decreasing with the decline in the number of malaria cases in many countries. Thus, the accuracy of microscopic examination of stained blood smears is not that high for many laboratories. Here, we provide evidence that XN-31 has the same performance as microscopy to assess parasitaemia. This excellent correlation between XN-31 and microscopy gives a strong rationale for the use of XN-31 for the assessment of parasitaemia. Additionally, XN-31 can also determine*Plasmodium*species as “*falciparum*” and “non-*falciparum*”. Acute determination of non-*falciparum*species is not available today. Moreover, XN-31 is an expensive method that will be possible to implement only in major health facilities. This problem of cost could be overcome given the excellent results of our repeatability study. One can imagine that healthcare facilities that did not have direct access to XN-31 will use conventional screening methods, including LAMP and RDTs, and will send their blood samples for diagnostic confirmation and parasitaemia assessment to laboratories where an XN-31 system is in use. Although XN-31 is an attractive method for malaria diagnosis, it is not possible to base the malaria diagnostic strategy on this method alone. Microscopy is still needed to confirm the identification of*Plasmodium*species and to describe complex diagnostic scenarios. This would also prevent any problems due to reagent shortage, machine breakdown, or computer issues. Automatic staining of blood smears combined with artificial intelligence (AI) for species identification and parasitaemia count appears to be the best partner for XN-31 in the near future [18,24]. Many teams or companies are working on this topic, but unfortunately, we are still far from commercial deployment of these AI machines [2].

In our study, we demonstrated that XN-31 has the technical requirements to change the diagnostic malaria strategy in well-equiped laboratories. Thus, it is of utmost importance to work with key players to make this transition possible. This task may also be supported by antimalarial stewardship programs [3].
